# Intercropping Rosemary (*Rosmarinus officinalis*) with Sweet Pepper (*Capsicum annum*) Reduces Major Pest Population Densities without Impacting Natural Enemy Populations

**DOI:** 10.3390/insects12010074

**Published:** 2021-01-15

**Authors:** Xiao-wei Li, Xin-xin Lu, Zhi-jun Zhang, Jun Huang, Jin-ming Zhang, Li-kun Wang, Muhammad Hafeez, G. Mandela Fernández-Grandon, Yao-bin Lu

**Affiliations:** 1 State Key Laboratory for Managing Biotic and Chemical Threats to the Quality and Safety of Agro-Products, Institute of Plant Protection and Microbiology, Zhejiang Academy of Agricultural Sciences, Hangzhou 310021, China; lixiaowei1005@163.com (X.-w.L.); lxx2026383726@126.com (X.-x.L.); zhijunzhanglw@hotmail.com (Z.-j.Z.); junhuang1981@aliyun.com (J.H.); zhanginsect@163.com (J.-m.Z.); wanglikun1314@sina.cn (L.-k.W.); drhafeez@webmail.hzau.edu.cn (M.H.); 2 Natural Resources Institute, University of Greenwich, Chatham Maritime, Kent ME4 4TB, UK; m.fernandez-grandon@greenwich.ac.uk

**Keywords:** aromatic plants, habitat manipulation, biological control, pest densities, natural enemy densities

## Abstract

**Simple Summary:**

Due to the harmful effects of pesticides on the environment and human health, alternative control methods have become more favored in vegetable pest management. Intercropping and natural enemy release are two widely implemented environmentally friendly control methods. In this study, the impact of sweet pepper/rosemary intercropping on pest population suppression was evaluated under greenhouse conditions and the effect of rosemary intercropping on natural enemy population dynamics was investigated. The results showed that intercropping rosemary with sweet pepper significantly reduced the population densities of three major pest species on sweet pepper, *Frankliniella intonsa*, *Myzus persicae*, and *Bemisia tabaci*, but did not affect the population densities of released natural enemies, predatory bug *Orius sauteri*, and parasitoid *Encarsia formosa*. Significant pest population suppression with no adverse effect on released natural enemy populations in the sweet pepper/rosemary intercropping system suggests this could be an approach for integrated pest management of greenhouse-cultivated sweet pepper.

**Abstract:**

Intercropping of aromatic plants provides an environmentally benign route to reducing pest damage in agroecosystems. However, the effect of intercropping on natural enemies, another element which may be vital to the success of an integrated pest management approach, varies in different intercropping systems. Rosemary, *Rosmarinus officinalis* L. (Lamiaceae), has been reported to be repellent to many insect species. In this study, the impact of sweet pepper/rosemary intercropping on pest population suppression was evaluated under greenhouse conditions and the effect of rosemary intercropping on natural enemy population dynamics was investigated. The results showed that intercropping rosemary with sweet pepper significantly reduced the population densities of three major pest species on sweet pepper, *Frankliniella intonsa*, *Myzus persicae*, and *Bemisia tabaci*, but did not affect the population densities of their natural enemies, the predatory bug, *Orius sauteri*, or parasitoid, *Encarsia formosa*. Significant pest population suppression with no adverse effect on released natural enemy populations in the sweet pepper/rosemary intercropping system suggests this could be an approach for integrated pest management of greenhouse-cultivated sweet pepper. Our results highlight the potential of the integration of alternative pest control strategies to optimize sustainable pest control.

## 1. Introduction

Due to the harmful effects of synthetic pesticides on the environment and human health, in addition to reduced efficacy due to resistance within pest populations, alternative control methods have become more favored in the framework of integrated pest management (IPM) [1]. Two widely implemented systems within IPM are the “push–pull” strategy and the introduction of biological control agents to achieve sustainable control [2,3]. The manipulation of insect behavior via plant volatiles is one of the key components of push–pull strategies [4,5,6]. Insects use plant volatiles to locate and recognize potential plant hosts for feeding and oviposition [7,8]. Accordingly, some non-host plants (e.g., aromatic plants) emit volatiles with repellent or deterrent properties as a defense against attack [9] and could be used to develop insect repellents, antifeedants, or insecticides [10,11]. Alternatively, non-host plants could disrupt host-plant finding and host-plant acceptance behavior by providing insects with a choice of green surfaces on which to land (host and non-host plant leaves), according to the ‘appropriate/inappropriate landings theory’ [12,13,14]. For these reasons, aromatic plants have been frequently used as intercrops to reduce pest damage to cultivated plants [15,16,17,18,19]. Intercropping aromatic plants could also increase the efficiency of biological control by attracting natural enemies to the area [20,21], providing food resources [15,17], or offering shelter and oviposition sites [21]. However, intercropping does not invariably result in an improvement in biological control [22,23]. For instance, in wheat-based intercropping systems, although pest abundance was significantly reduced, regulation through natural enemies was not necessarily enhanced [24]. Another study demonstrated that intercropping actively reduces the nocturnal biological control of aphids in a collard greens/parsley plants intercropping system [25]. Consequently, in order to optimize pest control in intercropping systems, the effects of intercropped plants on both pests and natural enemies should be evaluated and implemented on a case-by-case basis.

Rosemary (*Rosmarinus officinalis* L.) (Lamiaceae) is an aromatic plant mainly cultivated in the Mediterranean region. The plant, and its essential oil, are widely used for ornamental, culinary, cosmetic, and medicinal purposes [26,27,28]. The volatile compounds released by rosemary and its essential oils have been elucidated through several studies [29,30,31,32,33,34]. Although their composition varies among different studies [29,30,31,32,33,34], a common feature is that they all show α-pinene, eucalyptol (1,8-cineole), camphor, camphene, and verbenone as the most abundant compounds. The behavioral response of several pests to rosemary volatiles has been evaluated with an aim to develop an effective push–pull strategy [35,36]. Rosemary volatiles have been reported to be repellent to spider mites *Tetranychus urticae* Koch (Acari: Tetranychidae) [37], aphids *Myzus persicae* (Sulzer), and *Neotoxoptera formosana* (Takahashi) (Hemiptera: Aphididae) [38,39,40], whitefly *Bemisia tabaci* Gennadius (Hemiptera: Aleyrodidae) [32], thrips *Thrips tabaci* Lindeman and *Frankliniella occidentalis* (Thysanoptera: Thripidae) [31,41,42], the tea green leafhopper *Empoasca vitis* Gothe (Hemiptera: Cicadellidae) [33], and the tea geometrid *Ectropis obliqua* (Prout) (Lepidoptera: Geometridae) [34]. Consequently, rosemary has been used as an intercrop for reducing insect damage in the agricultural and horticultural systems in sweet pepper (*Capsicum annuum* L., Solanaceae) [43,44] and tea [*Camellia sinensis* (L.) O. Kuntze, Theaceae] fields [33,34]. A study in tea plantations found no effect of rosemary on generalist predator populations (spiders, ladybirds, and lacewings) [33]; however, beyond this example, previous studies have overlooked the implications rosemary may have on natural enemies with none exploring impacts on parasitoid success.

Sweet pepper (*Capsicum annuum* L., Solanaceae) is one of the most important horticulture crops globally [45]. It is susceptible to a range of pests, with thrips, whiteflies, and aphids considered the most important [45]. Currently, sustainable pest management in greenhouse-cultivated sweet pepper is mainly based on biological control [46,47]. Predatory bugs from the *Orius* genus are not only the most important natural enemies for thrips [48], but also contribute to the control of whiteflies [49] and aphids [50]. In addition to the *Orius* species, the parasitoid *Encarsia formosa* Gahan (Hymenoptera: Aphelinidae) has been used successfully to control whiteflies, including *Trialeurodes vaporariorum* Westwood and *Bemisia tabaci* Gennadius (Hemiptera: Aleyrodidae) [51,52]. Biological control is primarily used as part of an IPM approach and therefore its compatibility with other control methods could be key to sustainable suppression of the pest populations. In the present study, we combined rosemary intercropping and the release of biological control agents (predatory bug, *Orius sauteri* (Poppius) (Hemiptera: Anthocoridae), and parasitoid, *Encarsia formosa*) to control pests on sweet pepper. The impacts of these two control strategies on pest population suppression were evaluated. In addition, the effect of rosemary intercropping on natural enemies’ population dynamics was investigated. This study confirms the viability of this strategy for IPM in sweet pepper systems.

## 2. Materials and Methods

### 2.1. Field Setup

**Figure 1:**
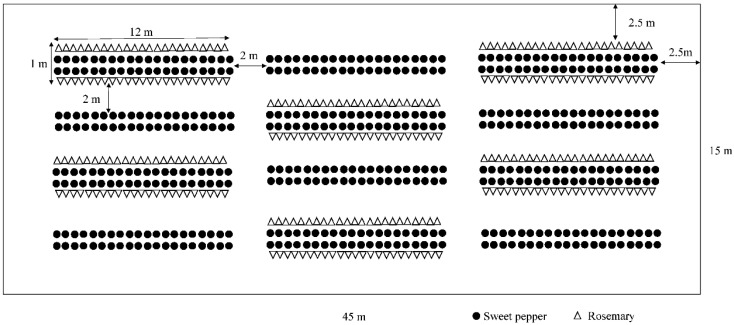
Schematic representation of the experimental greenhouse. The experimental area in the greenhouse was divided into 12 plots (1 m × 12 m), which were spaced 2 m apart from each other. In each plot, sweet pepper plants were separated by 40 cm and distributed among two rows spaced at 40 cm. Rosemary plants were planted at the outer edges of each intercropping plot, with a 30 cm distance from the sweet pepper and 40 cm in rows.

This study was conducted in a greenhouse (45 m × 15 m) at the Experimental Station of Zhejiang Academy of Agricultural Sciences, Jiaxing, Zhejiang, China (120°24′38.70″ E, 30°27′4.28″ N) in 2020. The experimental area in the greenhouse was divided into 12 plots (Figure 1). Plots (1 m × 12 m) were spaced 2 m apart from each other based on the results from previous studies [42,44]. The two planting systems, sweet pepper monoculture and sweet pepper/rosemary intercropping, were alternatingly represented throughout the glasshouse providing six plots of each (Figure 1).

Sweet pepper plants (*Capsicum annuum* var. Luojiaochengyan115) were sown in plastic nursery pots on 15 April 2020 in a separate greenhouse nursery and transplanted into the experimental greenhouse on 18 May 2020, when the seedlings were at the four to six true leaves stage. In each plot, sweet pepper plants were separated by 40 cm and distributed among two rows spaced at 40 cm. Rosemary (*R. officinalis* var. Zhili) seedlings (one to two years old, 15–20 cm in height) were bought from a nursery in Shouguang, Shandong, China. Rosemary plants were transplanted to the outer edges of each intercropping plot, with a 30 cm distance from the sweet pepper and 40 cm in rows. During the experiment, conventional fertilization and irrigation were carried out, and no insecticides, fungicides, or herbicides were used in the experimental area.

### 2.2. Natural Enemies Release

Predatory bug *Orius sauteri* adults and nymphs were purchased from Henan Jiyuan Baiyun Industrial Co., Ltd. (Jiyuan, China). *Orius sauteri* individuals were evenly released to all the plots at the second and third weeks (June 1 and June 8, to ensure the colonization of *O. sauteri* in the field) after transplantation at a release density of three individuals/m^2^ (plot area). Adults of the parasitoid *Encarsia formosa* were purchased from Woofutech Bio-control Co., Ltd. (Hengshui, China). *Trialeurodes vaporariorum* nymph cards with parasitoids were evenly released to all the plots when the whitefly density was five individuals (adults and nymphs) per leaf on sweet pepper leaves (June 29). The release density was 20 individuals/m^2^ (plot area).

### 2.3. Sampling of Pests and Natural Enemies on Sweet Pepper

The pest infestation samplings were conducted every week after transplantation until the end of the experiment (11 weeks). The major pests on sweet pepper in our greenhouse were thrips (*Frankliniella intonsa* (Trybom) (Thysanoptera: Thripidae)), aphids (*Myzus persicae*), and whiteflies (*Bemisia tabaci*). In each plot, three sweet pepper plants were randomly selected (at least four plants away from edges) and the number of *M. persicae* (adults and nymphs), *B. tabaci* (adults) and parasitoids *E. formosa* (adults) on five leaves at different directions of each plant were recorded. Because *F. intonsa* and predator bug *O. sauteri* were mainly distributed in sweet pepper flowers, 15 flowers (one to two flowers per plant, 10 to 15 plants in total) were randomly selected in each plot (at least four plants away from edges) and the number of thrips (adults and nymphs) and *O. sauteri* (adults and nymphs) were recorded. The mean number of individuals of each species per leaf or flower was calculated.

### 2.4. Statistical Analysis

Statistical analyses were conducted with SPSS statistical software (version 22.0) [53] and the R program (version 4.0.3) [54]. Since the majority of pests and natural enemies’ density data were not normally distributed according to non-parametric Kolmogorov–Smirnov tests in SPSS, differences in the densities of pests and natural enemies between sweet pepper monoculture and sweet pepper/rosemary intercropping treatments during the whole sampling duration (11 weeks) were determined using Generalized Linear Mixed Models (package ‘lme4’, function ‘glmer’) [55] with Poisson error distribution in the R program (*p* < 0.05). Treatment was included as a fixed factor, and sampling date as a random factor. Differences in the densities of pests and natural enemies on each sampling date between two cropping patterns were analyzed using Mann–Whitney *U* tests in SPSS (*p* < 0.05). Linear regressions were used to analyze the relationships between pest and natural enemy abundance. For *F. intonsa*, *M. persicae*, *B. tabaci*, and *O. sauteri*, the total abundance in each plot (in both treatments) was summed from the second week of *O. sauteri* release to the end of the experiments (June 8 to August 3), then linear regressions between the abundance of the three pest species and *O. sauteri* were analyzed. For *B. tabaci* and *E. formosa*, total abundance in each plot (in both treatments) was summed from the second week of *E. formosa* release to the end of the experiments (July 6 to August 3) with linear regressions between the abundance *B. tabaci* and *E. formosa* analyzed.

## 3. Results

### 3.1. Effect of Rosemary Intercropping on Population Dynamics of Pest Species

**Figure 2:**
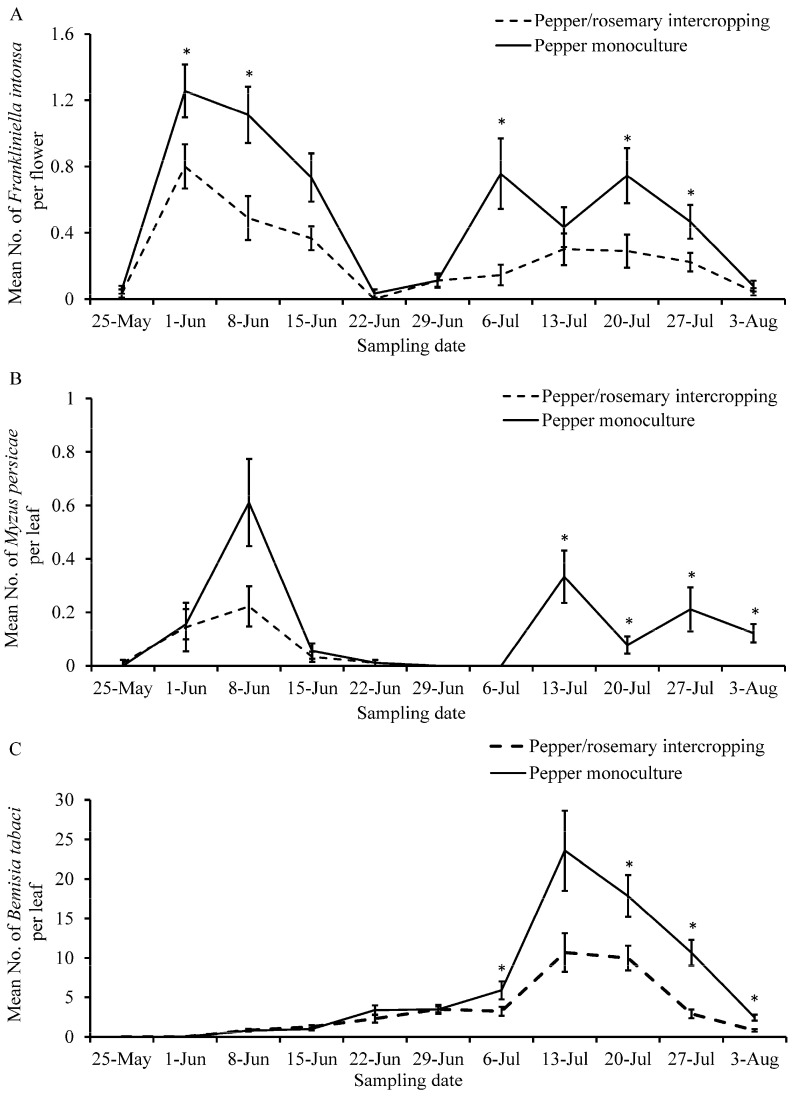
Mean population densities of pests on sweet pepper plants in sweet pepper monoculture and sweet pepper/rosemary intercropping treatments. (**A**) *Frankliniella intonsa*; (**B**) *Myzus persicae*; (**C**) *Bemisia tabaci*. Asterisks indicate significant differences in pest density at a given sampling date between sweet pepper monoculture and sweet pepper/rosemary intercropping treatments (*p* < 0.05).

*Frankliniella intonsa* densities throughout the sampling period were significantly lower in the sweet pepper/rosemary intercropping treatment compared to the sweet pepper monoculture treatment (*χ*^2^ = −9.469, *p* < 0.0001) (Figure 2A). Specifically, *F. intonsa* densities for the intercropped treatment were lower than those of the monoculture on June 1 (*U* = 3194.0, *p* = 0.008), June 8 (*U* = 2951.0, *p* < 0.0001), July 6 (*U* = 3531.5, *p* = 0.014), July 20 (*U* = 3380.5, *p* = 0.006), and July 27 (*U* = 3550.0, *p* = 0.050) (Figure 2A).

*Myzus persicae* densities throughout the sampling period were significantly lower in the sweet pepper/rosemary intercropping treatment compared to the sweet pepper monoculture treatment (*χ*^2^ = −7.307, *p* < 0.0001) (Figure 2B). Specifically, *M. persicae* densities for the intercropped treatment were lower than those of the monoculture on July 13 (*U* = 3510.0, *p* < 0.0001), July 20 (*U* = 3780.0, *p* = 0.013), July 27 (*U* = 3735.0, *p* = 0.007), and August 3 (*U* = 3555.0, *p* = 0.001) (Figure 2B).

*Bemisia tabaci* densities throughout the sampling period were significantly lower in the sweet pepper/rosemary intercropping treatment compared to the sweet pepper monoculture treatment (*χ*^2^ = −30.526, *p* < 0.0001) (Figure 2C). Specifically, *B. tabaci* densities for the intercropped treatment were lower than those of the monoculture on July 6 (*U* = 3364.5, *p* = 0.046), July 20 (*U* = 2970.5, *p* = 0.002), July 27 (*U* = 2689.0, *p* < 0.0001), and August 3 (*U* = 2213.0, *p* < 0.0001) (Figure 2C).

### 3.2. Effects of Rosemary Intercropping on Population Dynamics of Natural Enemies

**Figure 3:**
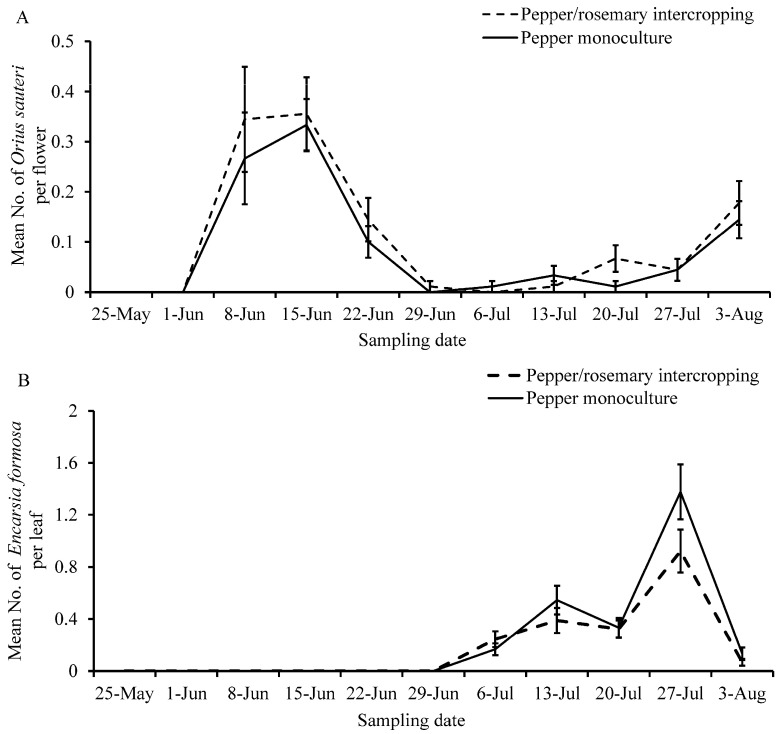
Mean population densities of natural enemies on sweet pepper plants in sweet pepper monoculture and sweet pepper/rosemary intercropping treatments. (**A**) Predator bug *Orius sauteri*; adults and nymphs of *O. sauteri* were released on June 1 and June 8. (**B**) Parasitoid *Encarsia formosa*; host nymph cards with *E. formosa* were released on June 29.

The density of the predatory bug, *O. sauteri*, throughout the whole sampling period was not significantly different between the intercropped and monoculture treatments (*χ*^2^ = 1.396, *p* = 0.163) (Figure 3A). The same result was also found for population densities of parasitoid, *E. formosa*, between the two treatments (*χ*^2^ = −2.472, *p* = 0.064) (Figure 3B).

### 3.3. Effect of Natural Enemy Release on Pest Densities and Pest-Natural Enemy Regressions

**Figure 4:**
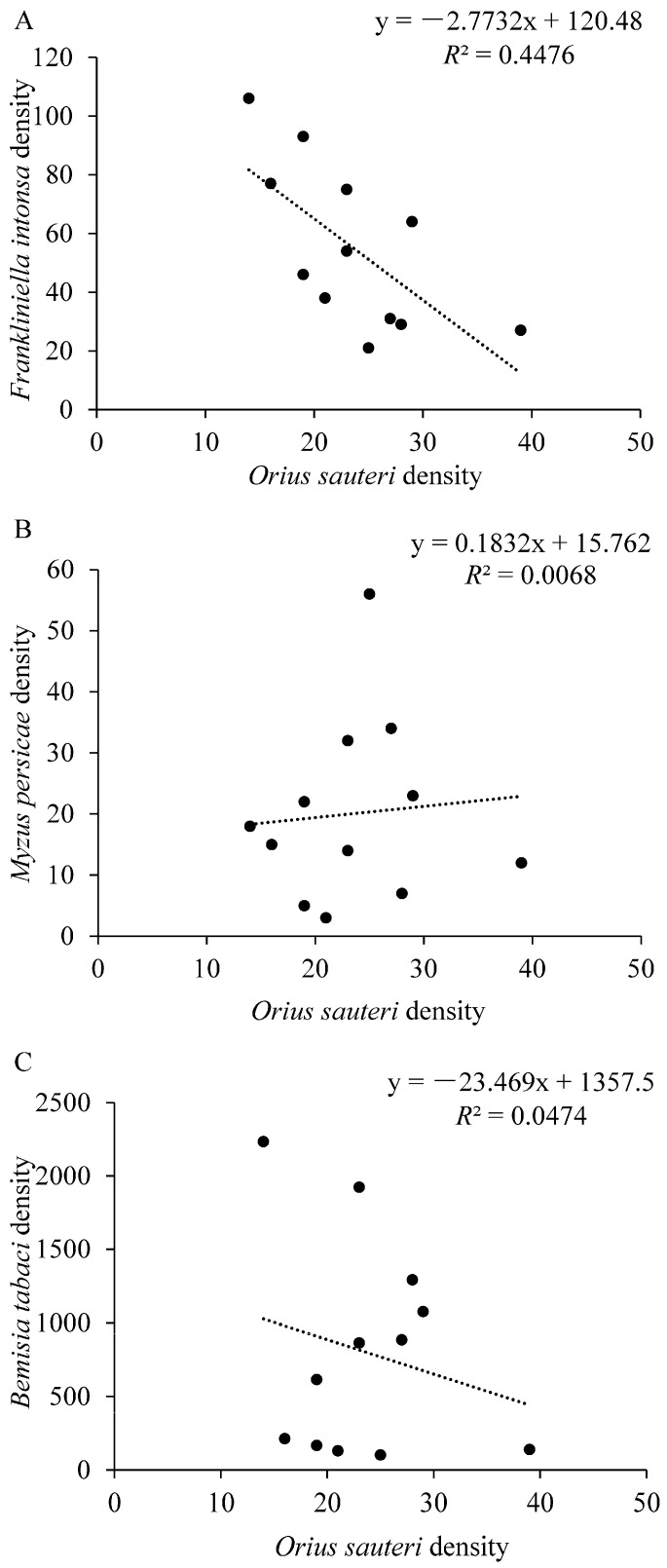
Linear regressions between *Frankliniella intonsa* (**A**), *Myzus persicae* (**B**), and *Bemisia tabaci* (**C**) and *Orius sauteri* densities. The densities of each species are the sums of 1620 observations.

**Figure 5:**
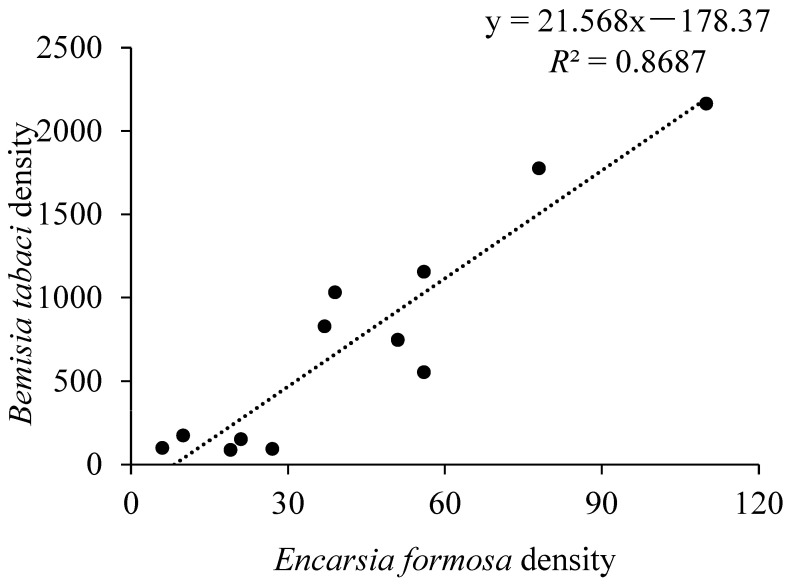
Linear regression between *Bemisia tabaci* and *Encarsia formosa* densities. The densities of each species are the sums of 1080 observations.

The number of *F. intonsa* decreased sharply after the release of *O. sauteri* (Figure 2A) and were negatively correlated with the densities of *O. sauteri* (*F*_1,11_ = 8.102, *p* = 0.017, *R*^2^ = 0.4476) (Figure 4A). Although the number of *M. persicae* decreased after the release of *O. sauteri* (Figure 2B), the densities of *M. persicae* and *O. sauteri* were not correlated (*F*_1,11_ = 0.069, *p* = 0.799, *R*^2^ = 0.0068) (Figure 4B). The density of *B. tabaci* was not correlated with the density of *O. sauteri* (*F*_1,11_ = 0.497, *p* = 0.497, *R*^2^ = 0.0474) (Figure 4C), but positively correlated with the density of the parasitoid *E. formosa* (*F*_1,11_ = 66.145, *p* < 0.0001, *R*^2^ = 0.8687) (Figure 5). The release of *O. sauteri* inhibited population growth of *B. tabaci* until June 29 (Figure 2C). When the population density of *O. sauteri* became low (June 29) (Figure 3A), the population density of *B. tabaci* started increasing dramatically (Figure 2C). The parasitoid, *E. formosa*, was released on June 29 and the density of *B. tabaci* started to decrease two weeks later (from July 13) (Figure 2C).

## 4. Discussion

Intercropping with rosemary significantly decreased the population density of *M. persicae* on sweet pepper, which is consistent with a previous field study by Ben Issa et al. [44]. Moreover, the population densities of two other pests, *F. intonsa* and *B. tabaci*, were also significantly suppressed by rosemary intercropping. The repellent chemical hypothesis, which states that non-host plant volatiles disrupt host location and feeding by herbivores, could explain why pest densities in the intercropped treatment were lower than in those in the sole crop [56]. It has been reported previously that rosemary volatiles were repellent to *M. persicae* both in the laboratory and screenhouse [38,40]. Rosemary volatiles are also repellent to *B. tabaci* [32] and more recent work has indicated repellence for three thrips species, including *F. intonsa* in laboratory-based olfactometer and host plant selection bioassays [42]. However, the persistent value of this repellent effect in field conditions needs further study. It is recognized that contributing factors are myriad and the volatile interaction between non-host plants and host plants might result in different behavioral responses in pests for different systems [57]. In addition, the release of semiochemicals from the intercrop plants can be affected by many factors, such as varieties, growth stages, and season [32,34,40]. Our results confirmed that rosemary is effective in suppressing aphid, thrips, and whitefly populations on sweet pepper in the field and could be a good candidate as a repellent intercrop plant.

Another possible mechanism responsible for lower pest densities in the intercropping system is the disruption of insect host-plant finding and acceptance behavior suggested by the ‘appropriate/inappropriate landings theory’ [12,14]. This theory suggests that it is just the number of alternative green objects (non-host plants) surrounding a host plant that reduces colonization by pest insects rather than the release of volatile chemicals that deter the pests from landing [13]. The theory is based on detailed studies of the cabbage root fly [*Delia radicum* L. (Diptera: Anthomyiidae)] and suggests that the complete system of host plant selection involves a three-link chain of events in which the first link is governed by cues from volatile plant chemicals, the central link by visual stimuli, and the final link by cues from non-volatile plant chemicals [12]. It is possible that in the intercropping treatment in this study, host-plant finding and acceptance were disrupted by a proportion of the pests landing on the rosemary (alternative green surfaces) instead of the sweet pepper plants. However, further studies on the behavior of these three pest species are needed to test this possible hypothesis.

It has been reported that increasing crop biodiversity, such as intercropping, can enhance pest natural enemies in agroecosystems [20]. However, the effect of intercrops on natural enemies varies when different intercrops are used. In our study, intercropping with rosemary did not affect the population densities of predatory bug, *O. sauteri*, or parasitoid, *E. formosa*, on sweet pepper. Similarly, it has been reported that rosemary intercropping did not affect the population densities of generalist predators in tea plantations [33]. Several possible reasons might contribute to the above results. No enhancement of natural enemy success in rosemary intercropping treatment in our study, or a previous study [33], might indicate that rosemary did not have any behavioral effect on natural enemies. Alternatively, rosemary volatiles might manipulate the behavior of natural enemies, as reported by Bennison et al. [41], who showed rosemary leaves and volatiles were repellent to predatory bug *Orius laevigatus* in an olfactometer. However, in this environment, it may be that herbivore-induced plant volatiles (HIPVs), which are known to attract natural enemies [58,59,60,61], are sufficiently detectable and take precedent over any attraction the natural enemies might otherwise have to rosemary. Further study on the behavioral response of *O. sauteri* and *E. formosa* to rosemary volatiles and sweet pepper HIPVs are required to elucidate the details of this interaction.

Our results showed that in both intercropping and monoculture treatments, the population densities of *F. intonsa* and *M. persicae* decreased as *O. sauteri* population density increased. Although we could not rule out other factors that contributed to pest population suppression because no control treatment without natural enemy releases was included in our study, predation of *F. intonsa* and *M. persicae* by *O. sauteri* could be the most likely reason. The population suppression of thrips and aphids by *O. sauteri* reported here is consistent with that of previous studies [62,63,64]. These results provide further evidence for the benefits of predatory bugs from the *Orius* genus as an effective control method for thrips and aphids [48,65]. Sequential release of *O. sauteri* and *E. formosa* was also effective in the control of *B. tabaci* in both intercropping and monoculture treatments. These results were similar to those of a previous study, in which the combination of *O. sauteri* and *E. formosa* was shown to effectively control *B. tabaci* in the greenhouse [66]. Our results showed the population decrease in *B. tabaci* occurred two weeks following the release of *E. formosa*. The reason might be that, unlike predators which immediately kill the host, such as a koinobiont parasitoids, *E. formosa* does not usually cause the immediate death of the host, requiring the host to be alive for the early stages of larval development [67]. In our study, *F. intonsa* density was negatively correlated with the density of *O. sauteri* regardless of the cropping system, which was consistent with the negative correlation between predator and prey in other studies [68,69]. However, in another study, the abundance of predator hoverfly larvae was positively correlated with the number of aphids [70]. Unlike the negative correlation between predator and prey in our study, *B. tabaci* density was positively correlated with the density of its parasitoid *E. formosa*. Different relationships between pests and natural enemies might be due to different host selection and foraging behavior of predators or parasitoids. Moreover, interactions between host plants, pests, and natural enemies vary considerably among different systems. Further studies on chemical communications and insect behavior manipulations in different plant–prey–predator or plant–host pest–parasitoid systems are needed.

Natural enemy release has been widely used in greenhouse pest control, unlike intercropping, which is more commonly used in open fields and orchards [16,17,33,68,71]. Less research has explored its possible use in greenhouse pest control. Our study showed that rosemary intercropping is feasible in the greenhouse because it can be successfully established under vegetable-growing conditions without additional farming practices being implemented. Although the intercropping of rosemary plants would compete for nutrients and/or water with target crop plants, they also provide economic value as ornamental, culinary, cosmetic, or medicinal plants [26,27,28], and could provide a minor additional revenue for growers. Furthermore, significant pest population suppression and the lack of adverse effect on natural enemies in the sweet pepper/rosemary intercropping system suggest the potential of this combination in the IPM framework. However, because field conditions were complicated, and our study was only conducted for one growing season, further investigations are needed to confirm the validity of the results. Moreover, additional studies are needed to investigate whether this is effective in different spatial configurations of the two plants and in open field settings. Nevertheless, enhanced pest suppression by combining the two alternative control strategies reported here provides a promising direction for improving sustainable pest management.

## Data Availability

The data presented in this study are available in article.
